# Primary breast T-cell lymphoma, unspecified, treated with autologous peripheral blood stem cell transplantation: A case report and literature review

**DOI:** 10.3892/ol.2013.1676

**Published:** 2013-11-11

**Authors:** QIQI GAO, XIUMING ZHANG, HUA XIANG, GUOPING REN, YULONG ZHENG

**Affiliations:** 1 Department of Pathology, The First Affiliated Hospital, School of Medicine, Zhejiang University, Hangzhou, Zhejiang 310003, P.R. China; 2 Department of Medical Oncology, The First Affiliated Hospital, School of Medicine, Zhejiang University, Hangzhou, Zhejiang 310003, P.R. China

**Keywords:** breast, T-cell lymphoma, immunohistochemistry, recurrence

## Abstract

The current study presents a case of primary T-cell lymphoma (PTBL), unspecified, in a 27-year-old female. The patient received chemotherapy [cyclophosphamide, epirubicin, vindesine and prednisolone (CHOP) and VP-16 plus CHOP (ECHOP)] and autologous peripheral blood stem cell transplantation, however, relapse occurred rapidly. The recurrent tumor exhibited increased levels of karyopyknosis and nuclear fragmentation and a higher Ki67 index compared with the primary tumor. No response to subsequent chemotherapy, including ECHOP and gemcitabine, dexamethasone and cisplatin, was observed. The patient succumbed to PTBL, unspecified, 18 months after the diagnosis. We hypothesize that autologous peripheral blood stem cell transplantation is ineffective for PTBL.

## Introduction

Breast non-Hodgkin’s lymphoma (NHL) is a rare entity, comprising <0.5% of all breast malignancies and ~0.7% of all NHL cases, in which secondary forms are more common than primary (1,2). Breast lymphomas are most commonly B-cell and occasionally T-cell types (3). T-cell lymphomas have a poorer prognosis than the B-cell type (4). Primary breast lymphoma commonly presents as a painless breast mass, which is similar to breast carcinoma (5). The right breast has been previously reported to be most frequently involved, however, the cause of this remains unknown (6). In the present case report, a rare case of primary T-cell breast lymphoma (PTBL) is described, including the histopathological and immunohistochemical observations of the primary and recurrent tumors and the clinical observations.

## Case report

### Patient presentation

A 27-year-old Chinese female presented with a left mammary mass to the First Affiliated Hospital, Hangzhou, China, in April 2010. There was no history of fever, weight loss, night sweats or other symptoms. The patient’s medical and family histories were unremarkable. Upon physical examination, a single non-tender mass was palpable at the upper outer quadrant of the left breast and an enlarged lymph node was palpable in the ipsilateral axilla. There was no cervical or inguinal lymphadenopathy. An ultrasound examination revealed a hypoechoic, non-cystic mass with ill-defined borders in the upper outer quadrant of the left breast, which measured 3.3×2.6 cm. Computed tomography (CT) scans of the abdomen, chest X-rays, a bone marrow aspiration and a biopsy revealed no significant observations. Laboratory data, including the total white cell count, lactate dehydrogenase (LDH), β2-microglobulin and immunoglobulin levels, were normal.

### Histopathological and immunohistochemical observations

**Figure 1:**
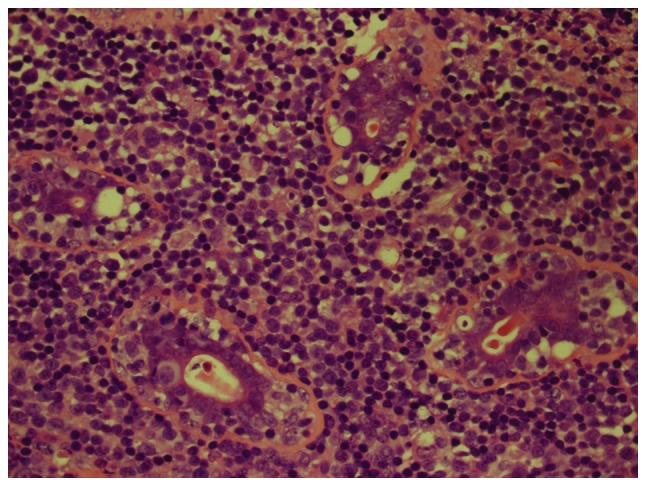
Hematoxylin and eosin staining. The tumor contains sheets of small and medium-sized atypical lymphoid cells, and infiltration into ducts and blood vessels (magnification, ×400).

**Figure 2:**
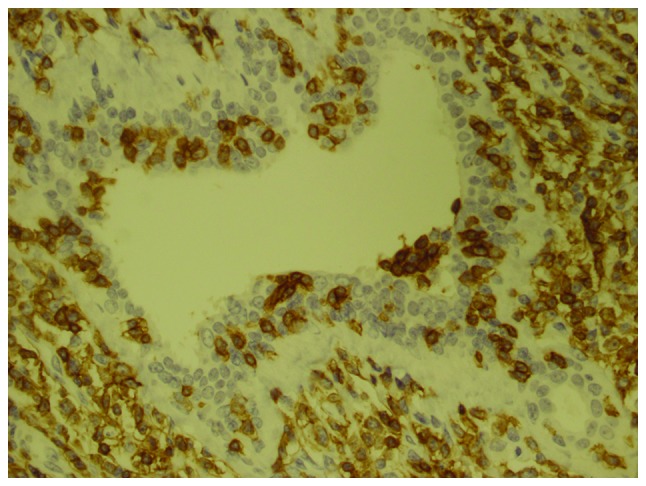
Immunohistochemical analysis performed on paraffin sections. Neoplastic cells show positive staining for the T-cell antigen, CD3 (magnification, ×400).

A segmental resection was performed. Examination of the hematoxylin and eosin staining revealed sheets of uniform, small and medium-sized, round, blue cells, and infiltration into ducts and blood vessels in certain regions was observed. Mitotic figures and a large number of eosinophils were observed (Fig. 1). A panel of antibodies was used for an immunohistochemical study, including CD2, CD4, CD43, CD3, CD5, CD8, p53, cyclin D1, cytokeratin, vimentin, desmin, myeloperoxidase (MPO), CD79a, CD7, CD30, anaplastic lymphoma kinase (ALK), CD34, CD10, CD56, granzyme B, B-cell lymphoma 2 (BCL2), BCL6, S100, terminal deoxynucleotidyl transferase (TDT), estrogen receptor (ER), progesterone receptor (PR) and paired box (pax)5. The tumor cells were markedly positive for CD2, CD4, CD43 and CD3 (Fig. 2) and weakly positive for CD5, CD8 and p53. Certain tumor cells were cyclin D1-positive. Reactivity for cytokeratin, vimentin, desmin, MPO, CD79a, CD7, CD30, ALK, CD34, CD10, CD56, granzyme B, BCL2, BCL6, S100, TDT, ER, PR and pax5 was negative. Based on these observations, the patient was diagnosed with PTBL, unspecified. A subsequent positron emission tomography (PET)/CT scan demonstrated multiple hypermetabolic foci in the left breast and the ipsilateral axillary lymph nodes.

### Drug administration

The patient was administered one cycle of CHOP (750 mg/m^2^ cyclophosphamide on day 1, 60 mg/m^2^ epirubicin on day 1, 1.4 mg/m^2^ vindesine on day 1 and 100 mg prednisolone on days 1–5, every 3–4 weeks). A chest CT scan revealed that the left breast was enlarged with multiple soft tissue masses. Subsequently, the patient received six cycles of ECHOP (120 mg/m^2^ VP-16 on days 1–3 plus CHOP every 3–4 weeks). Marked tumor regression was observed following the first cycle of ECHOP and a complete clinical response was achieved following the third cycle. Six months after diagnosis and two months after chemotherapy, the patient received an autologous peripheral blood stem cell transplantation. Following transplantation, a CT scan showed no evidence of recurrence.

### Recurrence

Six months following transplantation, two left mammary masses were identified by the patient. A PET/CT scan indicated an abnormal accumulation of fluorodeoxyglucose in the left breast, the left axilla and the right ilium, indicative of recurrence and metastasis. LDH levels were elevated to 315 U/l (normal limit, 91–250 U/l). β2-microglobulin and immunoglobulin levels remained within the normal limits. The patient underwent an excisional biopsy of the left breast. Histopathology showed that the recurrent tumor increased levels of karyopyknosis and nuclear fragmentation compared with the primary tumor. The positive expression rates of Ki67 in the recurrent tumor (~80%) were higher than in the primary tumor (~40%). The immunohistochemical results were consistent with those of the primary tumor. Subsequently, the patient received two cycles of ECHOP and one cycle of GDP (1000 mg/m^2^ gemcitabine on days 1 and 8, 40 mg dexamethasone on days 1–4 and 25 mg/m^2^ cisplatin on days 1–3, every 3–4 weeks). The recurrent tumor was resistant to the ECHOP and GDP regimen. The individual succumbed to PTBL, unspecified, 18 months following the diagnosis.

This study was approved by the institutional ethics committee of The First Affiliated Hospital, Zhejiang University. Informed consent was obtained from the patient’s family.

## Discussion

PTBL is an extremely rare and aggressive disease. The age range of patients with this disease is between 13 and 77 years old (3,7). The most common subtype of peripheral T-cell lymphoma is unspecified, accounting for ~50% of all cases. The standard diagnostic criteria to distinguish between primary breast lymphoma and a secondary form was outlined by Wiseman and Liao in 1972 (8). These include the following characteristics: i) Adequate pathological specimens; ii) mammary tissue and lymphomatous infiltration in close association; iii) no evidence of lymphomatous infiltrate with other lymphoma focus at the time of diagnosis, except for compromised ipsilateral axillary lymph node; and iv) no prior diagnosis of extra-mammary lymphoma. In the present case, the clinical features and histopathological and imaging observations fulfilled these four criteria. Thus, a diagnosis of unspecified PTBL was made.

To date, no clear clinical or radiological features have been described to distinguish primary breast lymphoma from any other type of infiltrating breast carcinoma, however, prominent lymph vessels in a patient with a breast mass and B symptoms (i.e. fever, night sweats and weight loss) must raise the suspicion of breast lymphoma, and the diagnosis may be excluded if calcifications or a desmoplastic reaction are present (9,10). Lymphoma must be included in the differential diagnosis of breast masses, since no pathognomonic radiological observations exist for its diagnosis (11). The key to the diagnosis of these cases remains as the requirement for adequate tissue from biopsy for histopathological evaluation and immunophenotyping. A core needle biopsy of the breast mass is sufficient to differentiate breast lymphoma from breast carcinoma. However, a segmental resection was performed on the patient. We consider that this was a pitfall in the treatment process of the patient in the present study.

The treatment modalities for PBTL have not been clearly defined (12). Bhele and Gujral described the case of a 26-year-old pregnant female with bilateral peripheral T-cell breast lymphoma successfully treated with CHOP chemotherapy (13). By contrast, in other cases, chemotherapy alone has been reported to be an inadequate treatment for this disease (14). In the current case, the patient exhibited a complete clinical response to the ECHOP regimen and subsequently received an autologous peripheral blood stem cell transplant. To the best of our knowledge, this is the first instance where a PTBL patient has been treated with this modality. However, the patient rapidly exhibited recurrence and metastasis following transplantation.

The recurrent tumor exhibited increased karyopyknosis and nuclear fragmentation compared with the primary tumor. Furthermore, the Ki67 index was higher. These changes are likely to imply that the recurrent tumor was more progressive than the primary one. Indeed, the relapsed tumor was not responsive to ECHOP and GDP.

Ann Arbor stage, International Prognostic Index (IPI), LDH and radiotherapy are important factors for the relapse-free survival of patients with lymphoma (14). According to the IPI, the current patient belonged to the moderate risk group. However, the patient relapsed after 12 months and succumbed to PTBL 18 months following diagnosis. The survival of the patient was shorter than the majority of previously reported PTBL cases. Aquino *et al* hypothesized that the overexpression of cyclin D1 may be an unfavorable prognostic factor in primary breast T-cell lymphoma, unspecified (15). In the current case, cyclin D1 was positive in certain regions, indicating that cyclin D1 may be a marker of poor prognosis. However, this must be confirmed in additional cases.

In conclusion, the optimal treatment for PTBL remains unknown. Autologous peripheral blood stem cell transplantation was ineffective to cure PTBL in the present case. The literature review indicated that a biopsy of any recurrent tumors is important and re-examination of the Ki-67 index may be useful for the prediction of response prior to the initiation of chemotherapy.
